# High-Precision Indoor VLP Scheme Based on the Synergy of SMO Multipath Suppression and Intelligent Algorithms

**DOI:** 10.3390/s26061826

**Published:** 2026-03-13

**Authors:** Yucheng Yang, Junyi Zhang, Shaohua Liu

**Affiliations:** School of Electronic Engineering, Beijing University of Posts and Telecommunications, Beijing 100876, China; 2023110454@bupt.cn (Y.Y.); liushaohua@bupt.edu.cn (S.L.)

**Keywords:** visible light positioning, multipath suppression, intelligent algorithms, sparse channel reconstruction

## Abstract

To address the issue that multipath effect severely restricts the performance of indoor visible light positioning (VLP) systems and multipath interference intensity varies significantly across different regions, this paper proposes a spatial adaptive multipath suppression scheme for the first time. At the transmitter, a hybrid transmission architecture of time division multiplexing (TDM) and direct current biased-orthogonal frequency division multiplexing (DCO-OFDM) is employed, providing ideal observation vectors for sparse channel modeling at the receiver through specialized pilot symbol design. At the receiver, a novel Spatial Adaptive–Main Path Energy Constraint–Orthogonal Matching Pursuit (SA-MPEC-OMP, SMO) algorithm is proposed to adapt to the spatial region characteristics with varying multipath intensities, enabling low-latency and accurate separation of Line-of-Sight (LOS) and Non-Line-of-Sight (NLOS) paths. Simulation results verify that the SMO algorithm achieves high main path extraction accuracy exceeding 90% in all regions, with its LOS energy ratio 2.7 to 3 times higher than that of the traditional OMP algorithm. Based on the results of the multipath suppression scheme, a high-precision 3D VLP scheme is proposed by integrating the SMO multipath suppression with intelligent algorithms. Specifically, a point classification model performs regional partitioning and dynamic threshold matching, while a height estimation model driven by LOS power extracted via SMO estimates the height of the target point. Finally, 3D coordinates are calculated using trilateration. Simulation results indicate that through the synergy of signal design and algorithm optimization, the proposed scheme achieves centimeter-level positioning across the entire space with a single positioning time of less than 18.7 ms, featuring strong multipath robustness and promising engineering application potential.

## 1. Introduction

### 1.1. Background and Motivation

In recent years, with the rapid development of Internet of Things (IoT) technology, intelligent terminals and artificial intelligence, the demand for high-precision indoor positioning in scenarios such as indoor navigation, industrial automation and medical equipment tracking has become increasingly urgent. Visible Light Positioning (VLP) technology has demonstrated great application potential in the field of indoor positioning due to its advantages such as no electromagnetic interference, sufficient spectrum resources and reusable infrastructure [1,2,3]. Current mainstream VLP methods are mainly divided into two categories: one is based on optical signal measurement principles, including classic positioning schemes such as Received Signal Strength Indicator (RSSI), Angle of Arrival (AOA) and Time Difference of Arrival (TDOA) [4,5,6]; the other is the multi-sensor fusion method, which combines auxiliary equipment such as Image Sensors (ISs) and Inertial Measurement Units (IMUs) to enhance positioning robustness [7,8,9]. Furthermore, with the continuous development of machine learning and neural network technologies, VLP schemes based on these methods have become one of the trends for solving positioning problems in complex indoor environments due to their strong non-linear fitting capabilities [10,11,12].

However, further review of relevant research results shows that existing VLP schemes still have limitations. For example, one of the current research hotspots in indoor VLP is positioning schemes based on RSSI fingerprint data and machine learning (ML) algorithms. Fite et al. [13] proposed a VLP scheme that fuses Principal Component Analysis (PCA) with Artificial Neural Network (ANN) regression. This scheme achieves high-precision indoor positioning by using PCA to reduce the dimensionality of high-dimensional RSS data and combining it with hyperparameter optimization such as the Adam optimizer and learning rate. Chang et al. [14] proposed a VLP system based on RSS preprocessing, Convolutional Neural Networks (CNNs) and Particle Swarm Optimization (PSO). This system achieved an average positioning error of 3.87 cm on a 2.5 m receiving plane. Although these research results have improved positioning accuracy and algorithm robustness, such methods require high costs for offline data collection and map construction. On the other hand, when positioning scenarios extend to three dimensions, a surge in the number of fingerprint points brings about a high computational complexity bottleneck, significantly increasing model training time [15,16]. Meanwhile, the multipath effect universally present in complex indoor environments has become a key bottleneck restricting the further improvement of VLP system performance [17]. For machine learning or neural network positioning methods based on RSSI, in a multipath environment, the RSSI value at the receiver is the result of the superposition of LOS signals and multiple NLOS signals. This leads to dynamic instability of RSSI fingerprint data and limits the generalization ability of the model. In addition, existing research on indoor multipath effect suppression mostly focuses on separate optimization at the signal or algorithm level [18,19], failing to fully leverage the synergistic advantages of signal design and receiving algorithms.

To address the aforementioned issues, this paper first proposes a pilot-aided spatial adaptive multipath suppression algorithm. By reconstructing the sparse channel within a compressive sensing framework, the proposed algorithm enables the low-latency and accurate separation of LOS and NLOS paths for each light source, which allows for the extraction of LOS power for the subsequent positioning process. To achieve 3D positioning, this study further integrates two lightweight intelligent algorithm models, specifically, a proposed height estimation model and a point classification model. While effectively controlling computational complexity, this scheme realizes centimeter-level positioning throughout the entire space and demonstrates a robust capacity for adapting to dynamic environments.

### 1.2. Contributions

The main innovations of this research can be summarized as follows:

This study adopts a hybrid transmission architecture of TDM and DCO-OFDM at the transmitter. Specifically, TDM ensures the time-slotted orthogonal transmission of signals from multiple light sources through time-slot partitioning. Furthermore, by designing pilot symbols using Shapiro–Rudin sequences with low peak-to-average power ratio (PAPR) characteristics, the system provides ideal observation vectors for constructing sparse channel models at the receiver. This allows the system to reconstruct sparse channel impulse response (CIR) with low computational latency and high efficiency, thereby suppressing multipath interference.

Traditional Orthogonal Matching Pursuit (OMP) algorithms rely solely on the maximum correlation between the path and the residual vector for path selection. In multipath scenarios, this often leads to hybrid NLOS paths forming temporary high correlations with the residual after superposition, which results in these paths being misidentified as valid and included in the support set, thereby causing support set contamination. Additionally, the iterative process cannot adaptively determine whether the reconstruction results align with the inherent characteristic of VLP channels where the main path energy proportion is prominent. To address this, this paper innovatively proposes a Main-Path Energy Constraint (MPEC) indicator to replace the single correlation judgment of traditional OMP and clearly define the dominant position of the direct path.

The proposed MPEC-OMP algorithm significantly suppresses support set contamination through the MPEC indicator. To further adapt to the differences in multipath intensity across indoor spaces and enhance main path extraction performance throughout the entire room, this study optimizes the spatial adaptability of MPEC thresholds. This paper implements a region-adaptive dynamic MPEC threshold mechanism and proposes the SA-MPEC-OMP (SMO) algorithm to achieve precise matching between MPEC thresholds and spatial multipath intensity characteristics, ensuring the accuracy of main path extraction across various multipath scenarios.

A 3D positioning scheme based on the synergy between SMO multipath suppression and intelligent algorithms is proposed. First, to overcome the difficulty of unknown height coordinates when extending indoor VLP to 3D scenarios, a height estimation model driven by pure LOS power is developed. The SMO algorithm is utilized to accurately separate the LOS component from the total received power, which resolves the problem of low discriminability in RSSI across different height planes caused by multipath interference. Second, based on the distribution of indoor multipath effects, a point classification model is employed to provide a clear basis for spatial partitioning to optimize MPEC thresholds. It also provides region labels for dynamic threshold matching during the positioning phase. Finally, based on the estimated height and the pure LOS power extracted by the SMO algorithm, the distance between the photodetector (PD) and each LED is calculated, and the 3D coordinates of the target point are solved using the Least Squares (LS) method.

The advantages of this scheme lie in its ability to accurately separate LOS power from the total link power with low computational latency. By relying on only two lightweight models for height estimation and region classification, the scheme achieves an optimal balance between positioning accuracy and complexity. Moreover, it demonstrates strong multipath robustness and dynamic environment adaptability. Notably, the system can extend from 2D to 3D positioning without additional hardware, showing significant potential for engineering implementation.

## 2. System Model

### 2.1. Indoor Visible Light Channel Model

**Figure 1:**
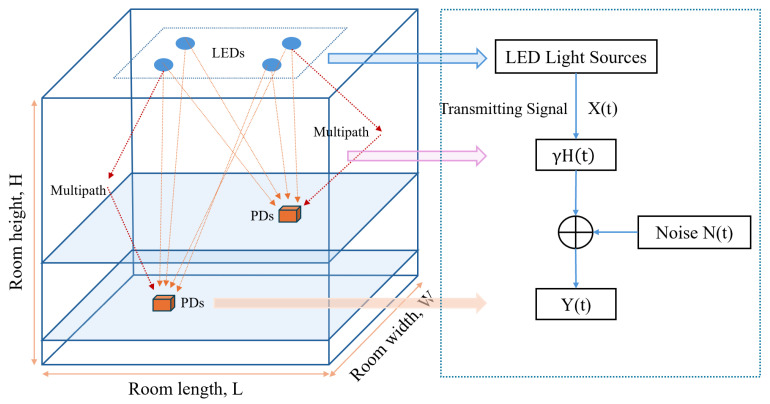
Geometric model and channel model of an LED-based VLP system (Adapted from [20]).

The indoor VLP system based on received signal strength (RSS) primarily consists of LED light sources, a visible light channel and a PD array, as shown in Figure 1. The length, width and height of the indoor space are denoted by *L*, *W* and *H*, respectively. Optical signals are reflected by the walls, ceiling and floor of the room. Multiple LED light source arrays are uniformly arranged on the ceiling, while the PD is located within the target positioning area.

The indoor visible light channel model can be expressed as

(1) $$Y\left(t\right)=\gamma \cdot \left(X(t)⊗H(t)\right)+N\left(t\right)$$

In the above equation, X(t) represents the intensity-modulated optical signal at the transmitter, Y(t) represents the photocurrent signal detected by the PD at the receiver, ⊗ denotes the convolution operation, γ is the responsivity of the PD, H(t) is the channel impulse response and N(t) represents the noise in the visible light communication (VLC) system.

**Figure 2:**
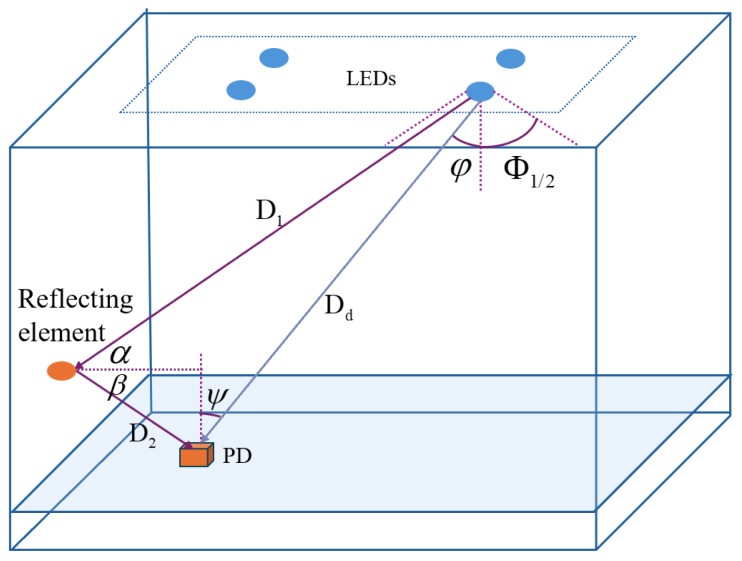
Indoor visible light LOS and NLOS channel models.

In indoor VLP systems, communication links can be divided into LOS links and NLOS links, as shown in Figure 2. The direct gain of the LOS channel can be expressed by the Lambertian radiation model as

(2) $$H_{\mathrm{LOS}}\left(0;S,R\right)=\left\{\begin{array}{cc} \frac{(m+1)A}{2\pi D_{d}^{2}}\cos^{m}\left(\phi \right)\cos\left(\psi \right)T_{s}\left(\psi \right)g\left(\psi \right), & 0\leq \psi \leq \Psi_{c}\\ 0, & \psi >\Psi_{c} \end{array}\right.$$

In the above equation, *S* and *R* represent the light source and PD, respectively; *m* is the Lambertian emission order; *A* is the active area of the PD; $D_{d}$ is the LOS distance from the LED source to the PD; ϕ and ψ are the angle of irradiance and the angle of incidence, respectively; $\Psi_{c}$ is the maximum field of view (FOV) of the PD; and $T_{s}\left(\psi \right)$ and g(ψ) represent the gains of the optical filter and the optical concentrator, respectively. The Lambertian emission order is determined by the semi-angle at half power of the LED source, $\Phi_{1/2}$. Its calculation formula is

(3) $$m=-\ln2/\ln(\cos\Phi_{1/2})$$

The positioning accuracy of an indoor VLP system is primarily determined by the received power $P_{r}$, which is the sum of the optical power received from both LOS and NLOS channels; therefore, it is also necessary to model the optical signal propagation along the NLOS channel. In the NLOS link, assuming the wall is a Lambertian diffuse reflecting surface, reflecting surfaces such as walls and ceilings can be approximated as many tiny reflecting elements dA. For a single reflection, the channel DC gain can be expressed as

(4) $$H{\left(0\right)}_{\mathrm{NLOS}}=\left\{\begin{array}{cc} \frac{(m+1)A}{2\pi D_{1}^{2}D_{2}^{2}}\rho \cos^{m}\left(\phi \right)dA\cos\left(\alpha \right)\cos\left(\beta \right)\cos\left(\Psi \right)T_{s}\left(\Psi \right)g\left(\Psi \right), & 0\leq \Psi \leq FOV\\ 0, & \Psi >FOV \end{array}\right.$$

In the above equation, ρ is the reflection coefficient. For multi-order reflections, this paper decomposes the NLOS link into three types of links: LED source to reflecting element, reflecting element to reflecting element, and reflecting element to PD.

The noise in the indoor VLP system is primarily Additive White Gaussian Noise (AWGN), which is composed of the superposition of shot noise and thermal noise. The background current $I_{bg}$ determines the ambient-light interference level.

Given the estimated height and LOS distances, the 3D coordinates are solved via standard LS-based trilateration.

### 2.2. Distance Calculation

In the VLC channel, when multi-path effects are not considered, the LOS link power can be expressed as

(5) $$P_{r}=\frac{(m+1)A}{2\pi D_{d}^{2}}\cos^{m}\left(\varphi \right)\cos\left(\psi \right)T_{s}\left(\psi \right)g\left(\psi \right)P_{t}$$

Based on Equation (5), if the LOS link received power is known, the distance between the PD and the LED light source can be expressed as

(6) $$D_{d}=\sqrt{\frac{\left(m+1\right)A\cos^{m}\left(\varphi \right)\cos\left(\psi \right)T_{s}\left(\psi \right)g\left(\psi \right)P_{t}}{2\pi P_{r}}}$$

When the LED plane is parallel to the PD plane, the transmitting angle is equal to the receiving angle, which is

(7) $$\cos\varphi =\cos\psi =\frac{h}{D_{d}}$$

In the above equation, *h* is the height difference between light source plane and receiver plane. After substituting Equation (7) into Equation (6), the distance can be expressed as

(8) $$D_{d}=\sqrt[{m+3}]{\frac{\left(m+1\right)AT_{s}\left(\psi \right)g\left(\psi \right)P_{t}h^{m+1}}{2\pi P_{r}}}$$

## 3. Pilot-Assisted Sparse Channel Modeling and SMO-Based Multipath Suppression for Indoor VLP

### 3.1. Pilot-Assisted TDM and DCO-OFDM Hybrid Transmission Architecture and Observation Vector Generation

**Figure 3:**
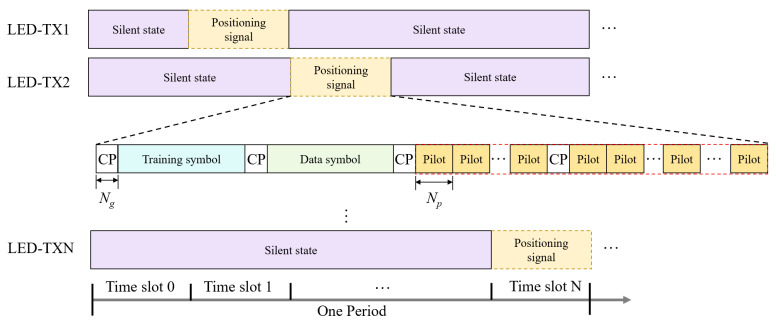
TDM Scheme and Signal Frame Structure for Indoor VLP.

To achieve cooperative positioning with multiple LEDs and a single PD, we employ a hybrid TDM + DCO-OFDM transmission framework, where TDM allocates orthogonal time slots to avoid inter-LED crosstalk, as illustrated in Figure 3. Each transmission period contains a global silent slot (for background-light sampling) and dedicated slots in which only one LED is active.

The positioning frame follows a “training–data–pilot” structure with a cyclic prefix (CP) appended to each segment to mitigate delay spread. Training symbols support timing synchronization; data symbols carry the LED identifier and transmitted optical power; and the pilot segment includes $N_{1}$ identical pilot symbols (length $N_{p}$) used for noise suppression via averaging.

**Figure 4:**
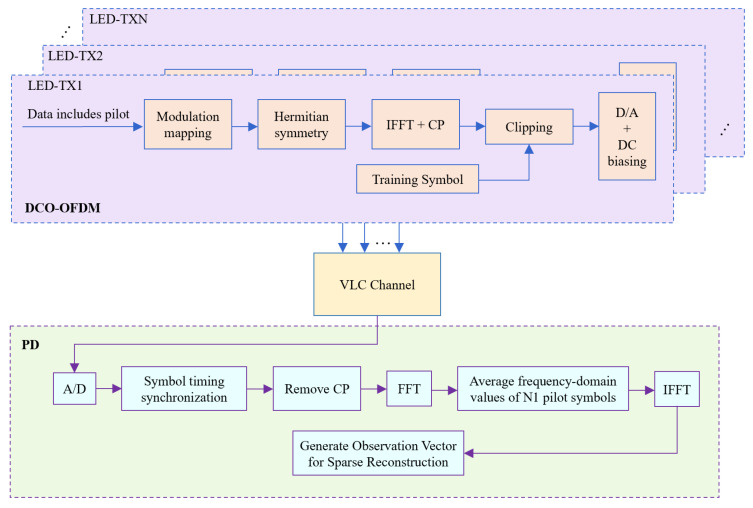
DCO-OFDM system.

For pilot design, Shapiro–Rudin sequences are adopted due to their low PAPR and favorable correlation properties for compressed-sensing-based reconstruction [21]. Figure 4 outlines the DCO-OFDM processing used to map these pilots into real-valued optical OFDM symbols.

At the receiver, the pilot segment contains $N_{1}$ repeated pilot symbols for each LED. For the same LED, these pilot observations are averaged to suppress AWGN, thereby generating a denoised observation vector ${\hat{Y}}^{i}$. The ${\hat{Y}}^{i}$ is then used as the input for the sparse CIR reconstruction in the following sections, from which the sparse CIR vector ${\hat{h}}^{i}$ is obtained. Once ${\hat{h}}^{i}$ is reconstructed, the LOS link power of the *i*-th LED is computed by

(9) $${\hat{P}}_{r,\mathrm{LOS}}^{\;i}=P_{r}^{\;i}\cdot {\hat{h}}_{\max}^{\;i}/\sum_{l=0}^{L-1}{\hat{h}}^{\;i}\left(l\right)$$

### 3.2. Sparse Channel Modeling

In indoor VLP, the received signal typically consists of one LOS component and only a few dominant reflected components. Therefore, the CIR vector ${\hat{h}}^{i}$ at the receiver exhibits clear sparsity in the time domain. Using the denoised observation vector ${\hat{Y}}^{i}$ defined in Section 3.1, the CIR estimation can be formulated as

(10) $${\hat{Y}}^{i}=\Phi {\hat{h}}^{i}+w^{i}$$

where the sensing matrix $\Phi \in R^{N_{p}\times L_{\max}}$ is formulated as a Toeplitz matrix composed of time-delay shifts of the pilot sequence, and $w_{i}$ denotes the AWGN. The recovery procedure in Section 3.3 develops a VLP-oriented recovery scheme that integrates an energy-consistency constraint, a region-adaptive mechanism, and reverse validation to robustly suppress multipath.

### 3.3. Optimized Sparse Channel Reconstruction Algorithm

Among sparse reconstruction methods, Orthogonal Matching Pursuit (OMP) is attractive for VLP due to its low complexity for lightweight hardware. However, in indoor multipath scenarios it may (i) falsely admit strong NLOS components early when they temporarily correlate with the residual; (ii) rely on non-adaptive residual thresholds that do not accommodate the spatial variation of multipath intensity (inner vs. edge/corner); and (iii) lack specific constraints to ensure the dominance of the LOS path.

To address these limitations, we propose the SA-MPEC-OMP (SMO) algorithm, featuring two core improvements. First, a time-delay weighted Main-Path Energy Constraint (MPEC) index replaces the single correlation criterion to evaluate main path dominance. Second, a region-adaptive threshold mechanism is designed and implemented. Specifically, a 3D point classification model is first utilized to partition the target positioning space according to the spatial distribution differences of multipath intensity. Subsequently, higher MPEC thresholds are applied to inner areas (weak multipath) for path purity, while lower thresholds are applied to edges/corners (strong multipath) to prevent LOS misjudgment. Furthermore, a rigorous and reproducible procedure for selecting the optimal MPEC thresholds for each region is detailed later in this section. Finally, based on these spatially matched thresholds, a reverse validation mechanism dynamically monitors support set updates and eliminates spurious paths, thereby guaranteeing absolute main path dominance and highly robust extraction across diverse indoor environments.

Assuming the estimated CIR vector at the current iteration is ${\hat{h}}^{i}$, the MPEC is defined as follows:

(11) $$MPEC=\frac{|{\hat{h}}_{\max}^{i}{\left(l\right)|}^{2}\cdot w_{\max}}{\sum_{l=1}^{L}{\left|{\hat{h}}^{i}\left(l\right)\right|}^{2}\cdot w_{l}}$$

where the weight $w_{l}$ corresponding to the *l*-th path is defined as

(12) $$w_{l}=\frac{1/\exp(\tau_{l}-\tau_{main})}{\sum_{k=1}^{N}1/\exp\left(\tau_{k}-\tau_{main}\right)}$$

If the current MPEC falls below the region-specific threshold $\tau_{MPEC}$, indicating a loss of main path dominance, reverse validation is triggered: the most recently added path is removed, and the MPEC is recalculated until $MPEC\geq \tau_{MPEC}$. Systematic simulations subsequently confirm that this spatial adaptive threshold mechanism significantly outperforms fixed thresholds in ensuring extraction accuracy and convergence stability across full-space scenarios.

**Figure 5:**
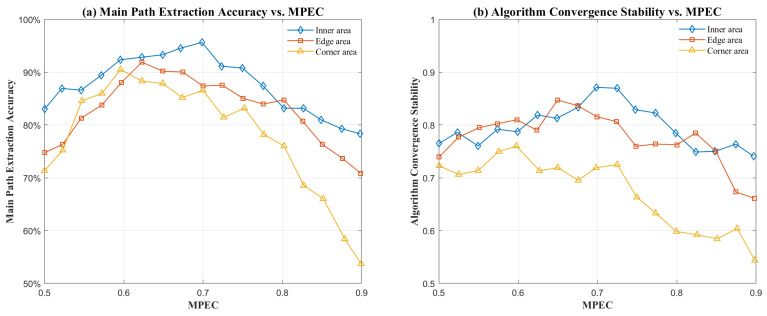
Impact of MPEC on the performance of the proposed algorithm. (**a**) Main path extraction accuracy; (**b**) convergence stability of the algorithm.

Figure 5 validates the effectiveness of the region-adaptive MPEC dynamic threshold. As illustrated in Figure 5a, owing to the weak multipath interference in the central region, the energy proportion of the main path is relatively high. When the MPEC threshold is set between 0.6 and 0.7, the main path extraction accuracy remains above 90%.

However, when the MPEC threshold exceeds 0.7, it becomes difficult for the main path proportion to meet the higher threshold requirements, leading to a gradual decline in accuracy. For edge regions with moderate multipath interference, the extraction accuracy peaks when the MPEC threshold is between 0.6 and 0.65. In corner regions, which suffer from strong multipath interference, the accuracy reaches its peak when MPEC is near 0.6. Although the extraction accuracy tends to decrease as multipath intensity increases, it still exceeds 90% for all three categories when the optimal MPEC threshold is adapted to each region. In contrast, using a global uniform MPEC threshold fails to maintain the main path extraction accuracy above 90% across the entire space, demonstrating the superiority of the proposed algorithm in terms of spatial adaptive thresholding.

Figure 5b further validates the convergence stability of the proposed algorithm. For the inner area, the algorithm achieves optimal convergence stability at an MPEC threshold of approximately 0.7. For the edge and corner regions, the best convergence stability is reached when the MPEC thresholds are near 0.65 and 0.6, respectively. By combining these results with the simulation findings in Figure 5a, the optimal MPEC thresholds for the inner, edge and corner regions are determined to be 0.7, 0.65 and 0.6. In summary, this study provides a methodology for determining the optimal MPEC parameters for various indoor regions: by fixing other parameters and adjusting only the MPEC threshold, we evaluate the main path extraction accuracy and convergence stability to select the optimal threshold for each region. This approach ensures both objectivity and reproducibility in the threshold selection process.

**Figure 6:**
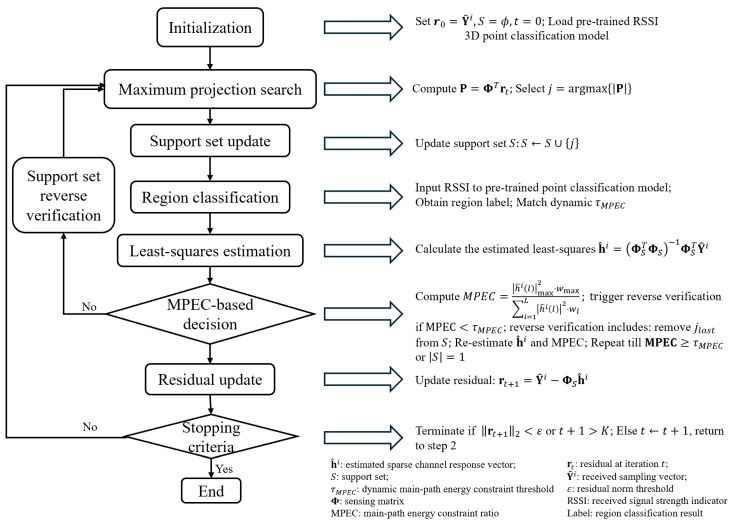
Flowchart of the proposed SMO algorithm.

**Figure 7:**
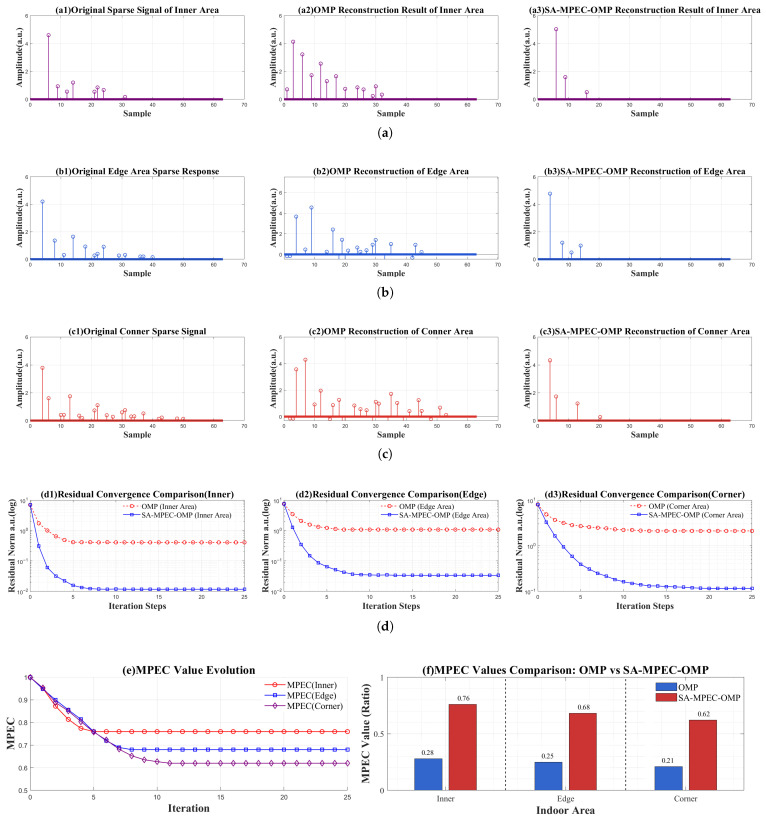
Performance comparison between regional OMP and SMO algorithms under multipath interference scenarios. (**a**) Sparse CIR reconstruction results in the inner region; (**b**) Sparse CIR reconstruction results in the edge region; (**c**) Sparse CIR reconstruction results in the corner region; (**d**) Residual convergence curves for different regions; (**e**) Iterative evolution curves of MPEC values; (**f**) Comparison of main path energy.

SMO Algorithm Flow and Performance Validation:

The complete execution flowchart of the proposed algorithm is illustrated in Figure 6. To verify the localization robustness and effectiveness of the proposed SMO algorithm in multipath scenarios, Figure 7 compares the performance of OMP and the proposed SMO algorithm for sparse channel reconstruction across different regions. As shown in Figure 7a–c, the reconstruction distortion of OMP increases with multipath intensity, leading to significant multipath contamination and several false amplitude components around the main path. Furthermore, some multipath components are significantly overestimated. Conversely, the proposed algorithm accurately restores the main path in all regions, characterized by clear peaks and consistent amplitudes. False multipath components are almost entirely suppressed due to the introduction of MPEC, which triggers a correction if the main path energy is insufficient. Figure 7d presents the residual convergence curves for both algorithms across different regions. Although the proposed algorithm requires more convergence time than OMP, it achieves lower stable residual values and superior reconstruction accuracy in the final iterations. Figure 7e illustrates the evolution of MPEC values using the proposed algorithm. It can be observed that the algorithm converges within approximately 10 iterations and maintains high threshold levels in each region. Notably, $\tau_{\mathrm{MPEC}}$ is a minimum acceptance threshold; thus, the converged MPEC after reverse validation can exceed it, explaining the difference between Figure 5 and Figure 7e. Figure 7f provides a quantitative comparison of the main path extraction capability. The MPEC of traditional OMP does not exceed 0.3 even in the inner region, whereas the MPEC of the proposed algorithm is 2.7–3 times higher than that of OMP across all regions. In summary, the proposed SMO algorithm effectively addresses reconstruction distortion and main path defocusing in traditional OMP under multipath scenarios. It demonstrates excellent performance across the entire space, providing an effective solution for multipath suppression and low-latency extraction of LOS power in VLP systems.

### 3.4. Performance Analysis of Indoor 2D Positioning Based on the SMO Algorithm

To evaluate the indoor positioning effectiveness of the proposed algorithm, this section compares it with the LS positioning method based on total received power. The performance is verified in an indoor 2D positioning scenario with a fixed height of h=1.0m. In this simulation, LS estimation algorithms using all four LED sources and the three LED sources closest to the PD are referred to as Benchmark Method 1 and Benchmark Method 2, respectively. The nonlinear LS estimation algorithm proposed in [22] is denoted by Benchmark Method 3. The proposed method utilizes the SMO algorithm to estimate the LOS link power and performs trilateration based on the calculated line-of-sight distances.

The positioning area is divided into a 5cm×5cm grid. At each grid point, the PD performs 20 positioning measurements to measure errors. The Cumulative Distribution Function (CDF) and Root Mean Square Error (RMSE) are adopted to evaluate positioning accuracy. The RMSE is calculated as follows:

(13) $$\mathrm{RMSE}=\sqrt{\frac{1}{N}\sum_{i=1}^{N}\left[{\left(x_{i}-{\hat{x}}_{i}\right)}^{2}+{\left(y_{i}-{\hat{y}}_{i}\right)}^{2}\right]}$$

where $\left(x_{i},y_{i}\right)$ and $\left({\hat{x}}_{i},{\hat{y}}_{i}\right)$ represent the true and predicted coordinates of the test points, respectively, and *N* denotes the total number of simulation runs for all sample points. This study uses MATLAB for modeling and simulation. The simulation software used is MATLAB R2024b and the simulations were conducted on a laptop equipped with an Intel Core i7-9750H CPU and 16 GB of RAM. No GPU acceleration was used in the simulations. The simulation parameters are listed in Table 1, most of which are identical or similar to those used in [23,24].

**Figure 8:**
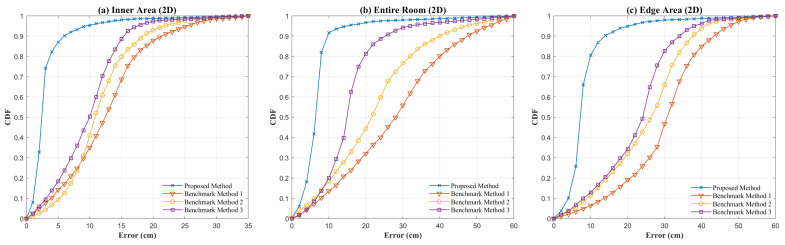
CDF comparison of four positioning methods across different indoor regions. (**a**) Inner Area (2D); (**b**) Entire Room (2D); (**c**) Edge Area (2D).

Figure 8 presents the CDF comparison between the proposed SMO-based positioning method and three benchmark methods in the inner area, the edge area and the entire room. Simulation results indicate that the CDF curves of the proposed method are significantly higher than those of the benchmark methods across all error distribution ranges, which reflects a substantial improvement in positioning performance. In the inner area, the proposed method achieves a much higher cumulative error probability within small error intervals (below 5 cm) compared to other methods. According to Table 2, its RMSE is only 2.7 cm, which is far superior to the 12.7 cm of Benchmark Method 3 (the best-performing benchmark in this region), representing an improvement of approximately 78.75%. In the more complex edge area where multipath interference is stronger, the proposed method maintains superior performance, with its CDF curve leading throughout. Specifically, its RMSE is 8.1 cm, while the RMSE of Benchmark Method 1 reaches 35.3 cm, resulting in a 77.05% improvement for the proposed method. Across the entire room, the positioning RMSE is 6.3 cm. This is the only solution among all evaluated methods that maintains the full-space RMSE below 10 cm.

The significant improvement in 2D positioning accuracy can be directly attributed to the physical-level error correction provided by the SMO algorithm. In multipath environments, the total optical power received by the PD is a superposition of the LOS and NLOS components. According to Equation (8), since the received power $P_{r}$ is located in the denominator, directly utilizing the multipath-inflated total power inevitably leads to an underestimated distance estimation. By dynamically extracting the pure LOS power, the SMO algorithm structurally eliminates this distance underestimation at the physical link level, thereby fundamentally ensuring the accuracy of the 2D positioning coordinates.

To further evaluate the precision and stability of the proposed scheme during repeated positioning, we calculated the standard deviation and normalized standard deviation (NSD) of the positioning errors in the 2D experiment, as presented in Table 3. Unlike the positioning accuracy reflected by RMSE and average error, the standard deviation and normalized standard deviation primarily indicate the dispersion of multiple measurements and the inherent stability of the system. They are defined as follows:

(14) $$\mu =\frac{1}{N}\sum_{i=1}^{N}e_{i},\;\sigma =\sqrt{\frac{1}{N-1}\sum_{i=1}^{N}{\left(e_{i}-\mu \right)}^{2}}$$

(15) $$\mathrm{NSD}=\frac{\sigma }{\mu }$$

where $e_{i}$ denotes the positioning error of the *i*-th measurement among *N* repeated tests at the same location, σ is the standard deviation and NSD is the normalized standard deviation.

Simulation results demonstrate that the standard deviation in the inner area is only 0.65cm with an NSD of 0.248, indicating a highly concentrated error distribution and stable positioning output. When evaluating the entire room, the standard deviation is 1.79cm and the NSD is 0.296, maintaining a low overall fluctuation. In the edge area, the standard deviation increases to 2.70cm and the NSD reaches 0.353, reflecting a relatively higher degree of error dispersion and increased fluctuation. Overall, the proposed scheme maintains a low NSD across different regions, demonstrating excellent positioning precision and stability.

**Figure 9:**
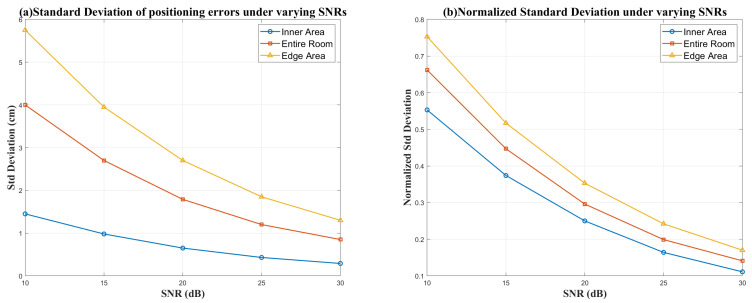
Standard deviation and normalized standard deviation of 2D positioning errors under varying SNRs in different regions. (**a**) Standard deviation; (**b**) normalized standard deviation.

As shown in Figure 9, we further analyze the standard deviation and normalized standard deviation of 2D positioning errors under varying signal-to-noise ratios (SNRs). As the SNR increases from 10 dB to 30 dB, the standard deviations across all three regions consistently decrease. This indicates that the fluctuations become smaller and the outputs become more stable at higher SNRs. Specifically, the standard deviation in the inner area gradually drops from 1.45 cm to 0.29 cm, while in the edge area, it decreases from 5.75 cm to 1.30 cm. Meanwhile, the NSD also continuously declines as the SNR increases. The NSD in the inner area drops from 0.553 to 0.111; in the edge area, it drops from 0.753 to 0.170. These results demonstrate that the proposed scheme maintains robust positioning precision across different spatial regions, with precision improving significantly as the SNR increases.

Notably, as the SNR continues to rise, the rate of decrease for both standard deviation and NSD gradually slows down. This suggests that at higher SNRs, the error dispersion approaches a stability floor mainly determined by the multipath distribution and geometric conditions of the scenario. Consequently, further increasing SNR provides limited additional gains in precision.

## 4. Proposed 3D VLP Scheme

**Figure 10:**
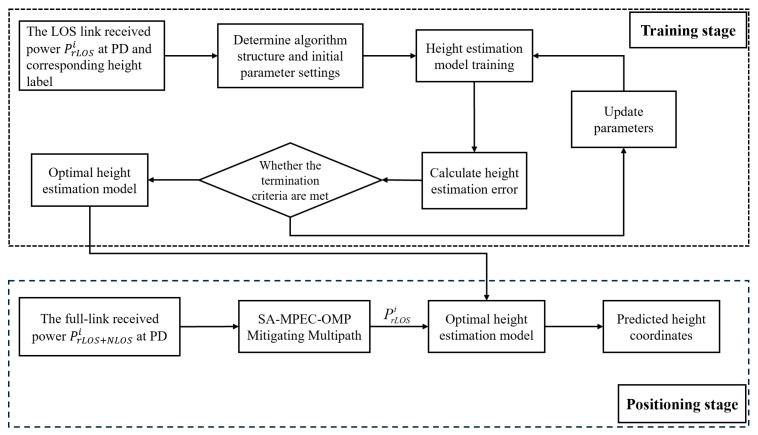
Training and testing flowchart of the height estimation model.

According to Equation (8), when the received optical power intensity is known, the calculation of the distance between the test point and the light source depends on the height difference between the PD and the light source plane. However, in indoor 3D positioning scenarios, the height of the test point is unknown, making it impossible to calculate the distance directly. Therefore, this paper proposes a height estimation scheme that combines SMO multipath effect suppression with a lightweight intelligent algorithm to investigate the mapping relationship between the PD height and the RSSI received from different LEDs. The constructed height estimation model is used to predict the *z*-coordinate of the test point while simultaneously addressing the distance calculation problem. Figure 10 illustrates the flowchart of the proposed height estimation model. It should be emphasized that during the model training phase, the input data consists of the LOS link power received from each LED. During the testing phase, the proposed SMO algorithm suppresses the multipath components from the total link power during preprocessing, enabling low-latency extraction of LOS power for height estimation.

The height range of the positioning area is defined as $H\in [H_{d},H_{u}]$, which is uniformly divided into $N_{H}$ segments with an interval of $d_{H}$, that is $N_{H}=\left(H_{u}-H_{d}\right)/d_{H}$. The set of height planes is $\left\{H_{1},H_{2},\ldots ,H_{N_{H}}\right\}$, where the *n*-th height plane is defined as $H_{n}=H_{d}+n\times d_{H}$, for $n\in \{1,2,\ldots ,N_{H}\}$. The corresponding set of height labels is $\left\{1,2,\ldots ,N_{H}\right\}$. Given that the estimated height is a continuous value, this study adopts a regression-based approach for training rather than multi-classification. Table 4 presents the performance comparison of the intelligent algorithms used for height estimation.

**Figure 11:**
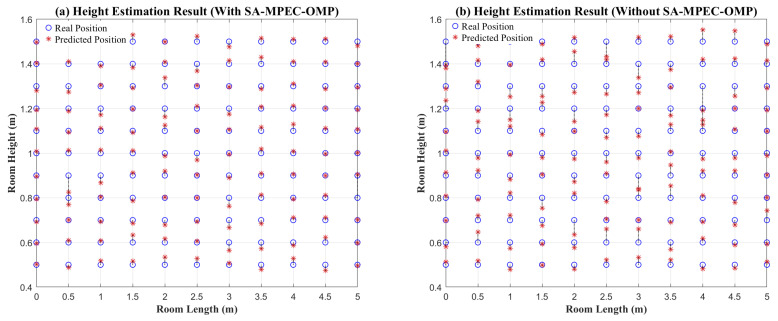
Height estimation result. (**a**) Height estimation results based on SMO multipath suppression; (**b**) height estimation results based on total link power.

Based on the height estimation results, the Sparrow Search Algorithm-optimized Support Vector Regression (SSA-SVR) model, which achieves the minimum height estimation error, is selected for the subsequent localization process. Figure 11a shows the height estimation results for the room diagonal plane, with a maximum error of 13 cm and an average error of 2.57 cm. Figure 11b displays the height estimation results of SSA-SVR based on the total link power, where the maximum error is 26.1 cm and the average error is 3.34 cm. The larger height estimation errors are primarily concentrated in the center of the room. This is because the center area is less affected by multipath effects, resulting in similar received power levels across different height planes, which reduces the discriminability of the model. It is important to note that while SSA-SVR achieves the lowest mean height-estimation error in our simulations, its margin over PSO-SVR is relatively small. Across repeated simulations with different random seeds and data splits, SSA-SVR and PSO-SVR show comparable performance with occasional rank swapping. Therefore, SSA-SVR is used as the default setting in this work, while substituting PSO-SVR does not lead to a material change in the end-to-end 3D positioning performance.

The substantial improvement in height estimation accuracy stems from resolving the feature confusion caused by multipath interference. According to Equation (8), accurate 3D distance calculation highly depends on the estimated height *h*. However, when the received power on adjacent horizontal planes is contaminated by multipath components, the spatial distinguishability of the Received Signal Strength Indicator (RSSI) is severely degraded, easily causing intelligent models to confuse the RSSI–height mapping relationship. By utilizing the pure LOS power extracted by the SMO algorithm as the input feature, the proposed method structurally eliminates this non-linear feature confusion. Consequently, combining the high-precision height prediction *h* with the accurately extracted LOS power ensures the precise calculation of the true 3D LOS distance.

**Figure 12:**
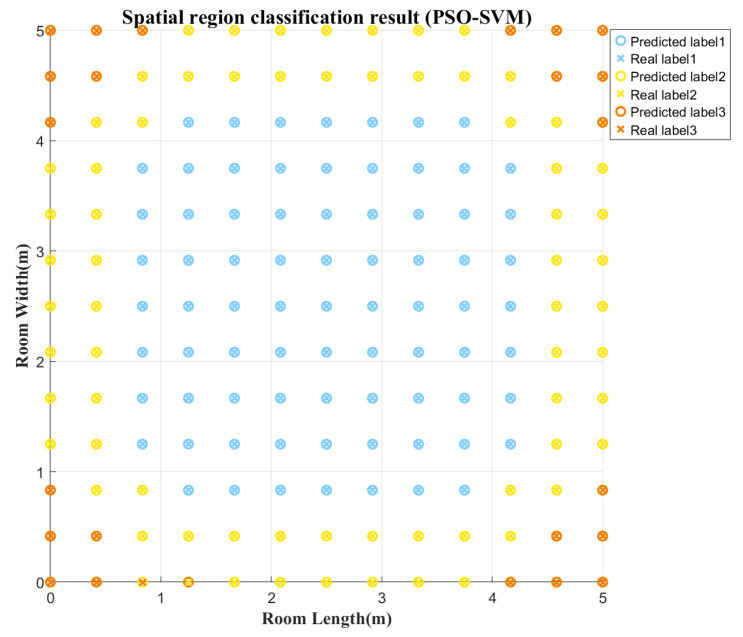
Prediction results of the PSO-SVM point classification model.

Since the proposed SMO algorithm utilizes spatially adaptive MPEC thresholds to suppress multipath interference, the region of the test point must be predicted during positioning to match the corresponding MPEC threshold of that region. To achieve spatial region estimation, this paper adopts the Particle Swarm Optimization algorithm optimized-Support Vector Machine (PSO-SVM) algorithm as a 3D point classification model. By utilizing the ratio of the first-order reflection to the LOS channel gain, $H_{1}/H_{0}$, the indoor space is divided into central, edge and corner regions. The model takes the RSSI of the test point as input and outputs the corresponding region label. Figure 12 illustrates the point classification results for a 1.2 m plane, with only one misclassified point at the boundary between the corner and edge regions. Table 5 shows the point classification results for three indoor height planes, where the classification accuracy exceeds 99% in all cases, verifying the effectiveness of the proposed classification model.

**Figure 13:**
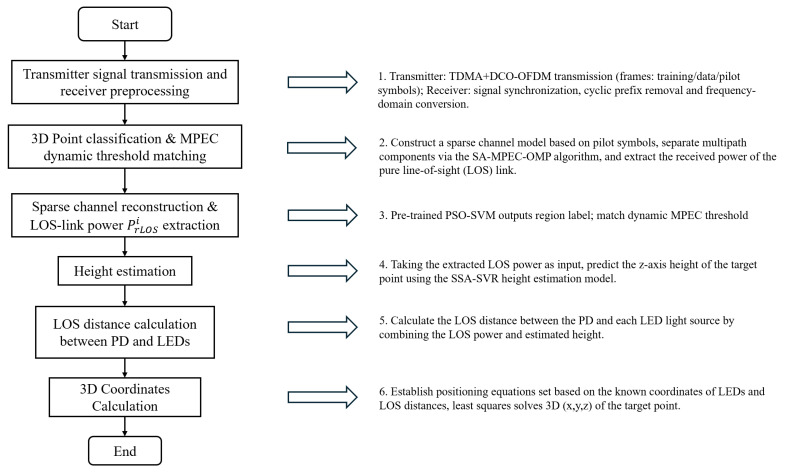
Overall flowchart of the proposed 3D VLP scheme.

**Figure 14:**
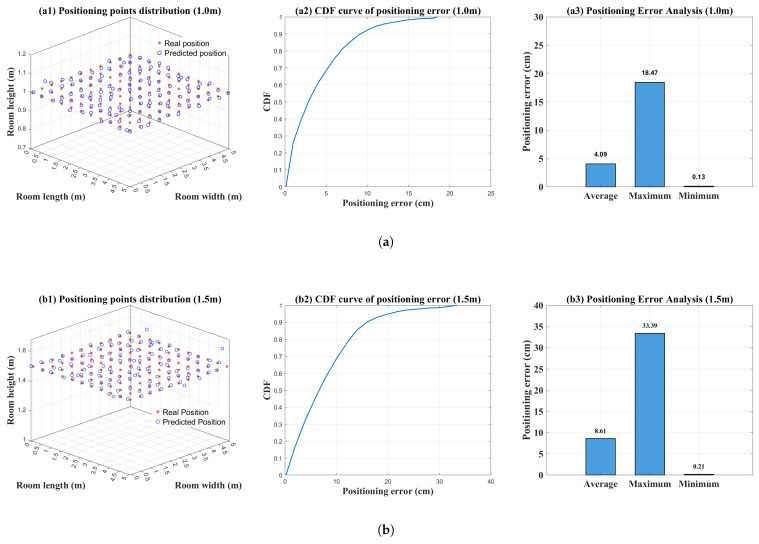

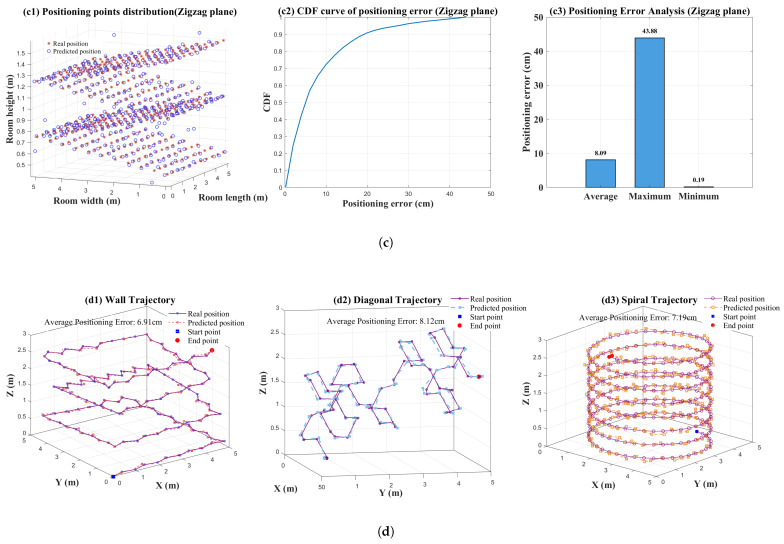
Positioning results for various test sets. (**a**) 1.0 m plane; (**b**) 1.5 m plane; (**c**) zigzag plane; (**d**) indoor trajectories.

This section proposes an indoor 3D VLP scheme based on the synergy of SMO-based multipath suppression and intelligent algorithms. Its complete positioning workflow is illustrated in Figure 13. To evaluate the performance of the proposed scheme, the average positioning error, maximum positioning error and CDF are utilized as evaluation metrics. Detailed simulation results are presented in Figure 14.

### 4.1. Simulation Results and Analysis of the Proposed Indoor 3D VLP Scheme

Figure 14a–c illustrate the localization results for the 1.0 m plane, 1.5 m plane and zigzag plane, respectively. Simulation results indicate that for the 1.0 m plane, the positioning error for 90% of the points is within 10 cm. For both the 1.5 m and zigzag planes, 90% of the positioning errors are within 20 cm. Although the heights of the target points in the zigzag plane alternate and fluctuate, leading to rapid variations in the channel environment that challenge the algorithm’s anti-interference capability and environmental adaptability, the proposed SMO algorithm combined with the height estimation scheme effectively maintains height errors within a small range. This reduces the error in calculating the LOS distance between the test point and the light source. The average positioning error for the zigzag plane is only 8.09 cm, which even outperforms the 8.61 cm achieved for the 1.5 m plane. The error in the 1.5 m plane primarily stems from its central location in the room, where the small variance in optical power distribution leads to slightly larger height estimation errors, subsequently affecting the distance calculation. The average positioning error for the 1.0 m plane is only 4.09 cm and the average errors for all three planes are within 10 cm.

Considering the terminal movement and position changes are random in practical scenarios, dynamic points were simulated and tracked within the room, resulting in the three trajectory plots shown in Figure 14d: wall-following, random diagonal and indoor spiral movements. The simulation results show that the average positioning error for all three trajectories is less than 9 cm. Combined with the results from the 1.0 m, 1.5 m and zigzag planes, the proposed 3D positioning scheme demonstrates the capability to achieve centimeter-level positioning accuracy.

To further evaluate the precision and stability of the proposed 3D positioning scheme during repeated measurements, we calculate the standard deviation and normalized standard deviation (NSD) of the 3D positioning errors across different indoor regions. The results are presented in Table 6.

Similar to the 2D case, the precision of 3D positioning exhibits clear spatial distribution characteristics. In the inner area with weak multipath interference, the 3D positioning standard deviation is only 1.67 cm and the NSD remains at a low level of 0.281. This indicates high stability in both height estimation and 3D coordinate calculation within this region. In the edge area, as multipath interference increases, the standard deviation and NSD rise to 3.33 cm and 0.366, respectively. In the corner area where multipath interference is the strongest, the standard deviation reaches 4.64 cm with an NSD of 0.427, reflecting a relative decrease in stability. This is primarily because the strong multipath effects in the edge and corner areas not only interfere with the extraction of LOS power by the SMO algorithm but also propagate errors to the height estimation model. Consequently, the dispersion of 3D calculation results increases compared to the 2D scenario.

Nevertheless, even in the corner area with the most severe multipath interference, the 3D positioning standard deviation of the proposed scheme is controlled within 5 cm. These results fully demonstrate that the proposed scheme maintains excellent positioning precision and strong system robustness in 3D indoor environments.

### 4.2. Overall System Architecture and Runtime Overhead Analysis

**Figure 15:**
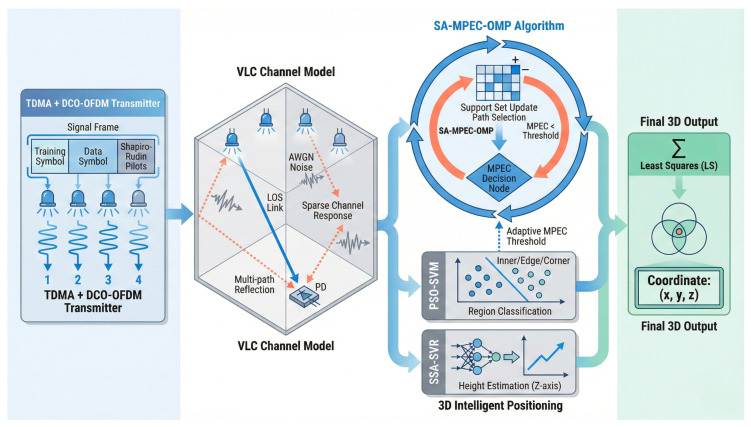
Overall architecture of the proposed VLP system.

The overall architecture of the proposed VLP system is shown in Figure 15, consisting of three modules: transmitter, visible light channel and receiver. The transmitter adopts a hybrid TDM + DCO-OFDM framework. Multiple LED sources achieve orthogonal transmission via TDM. Shapiro–Rudin sequences are selected for pilot symbols in the signal frames, combined with Hermitian symmetry for optical modulation. The optical channel includes LOS and NLOS paths, where reflections from walls and ceilings create multipath components. The receiver uses a PD array to convert optical signals into electrical signals. After preprocessing, a sparse channel model is constructed using pilot symbols. The SMO algorithm efficiently separates the main path from multipath components to extract LOS link power. Finally, the point classification model identifies the region label to match the MPEC threshold, the height estimation model obtains the *z*-coordinate and the LS algorithm completes the 3D coordinate solution. Through the synergetic optimization of signal design, multipath suppression, height estimation and spatial adaptation, the proposed architecture achieves high-precision positioning in complex indoor environments.

**Figure 16:**
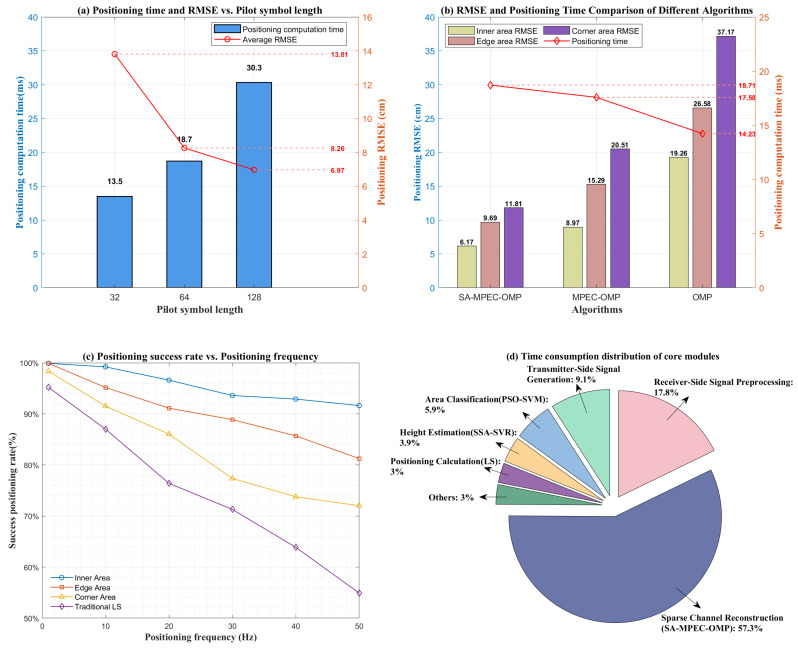
Analysis of system computational complexity and execution efficiency. (**a**) Impact of pilot symbol length on positioning performance and computational latency; (**b**) analysis of positioning performance and computational latency for three multipath suppression algorithms across different areas; (**c**) effect of positioning update rate on the positioning success rate of the proposed scheme in various regions; (**d**) processing time distribution of the core modules in the proposed positioning scheme.

Although the proposed indoor VLP scheme achieves high-precision positioning and dynamic adaptability, practical implementation requires a balance between performance and computational cost. Thus, this study quantitatively evaluates the system’s overhead and computational efficiency. Figure 16a illustrates the relationship between pilot symbol length, calculation time and RMSE. As the pilot length increases from 32 to 64, the calculation time rises from 13.5ms to 18.7ms. Meanwhile, the RMSE drops from 13.81cm to 8.26cm, which is a 40.2% reduction, significantly enhancing accuracy while maintaining high computational efficiency. When the pilot length further increases to 128, the RMSE only slightly decreases to 6.97cm, but the calculation time exceeds 30ms, leading to a notable latency degradation. Overall, setting the pilot symbol length to 64 provides the optimal balance between positioning accuracy and computational latency.

Figure 16b further compares the performance and time overhead of the VLP scheme using the proposed SMO algorithm against two comparative algorithms across different areas. In corner areas with the strongest multipath interference, the SMO algorithm maintains a high accuracy with an average RMSE of 11.81cm. This represents error reductions of 42.4% and 68.2% compared to MPEC-OMP (20.51cm) and OMP (37.17cm), respectively. Regarding time overhead, the comparative methods reduce calculation time by 6.04% and 23.95% compared to the proposed method. However, the proposed scheme’s positioning time remains within the 20ms low-latency threshold. The accuracy gains significantly outweigh the increased computational cost, achieving the optimization goal of “trading moderate complexity for significant improvements in positioning performance”.

Figure 16c shows the variation in the positioning success rate with positioning update rate for the proposed scheme across different regions. It also provides a comparison with the LS method based on total link Received Signal Strength. Simulation results indicate that the positioning success rate in each region remains above 85% even at a positioning update rate of 20Hz. This performance demonstrates strong feasibility for real-time deployment in most indoor positioning applications. Figure 16d illustrates the execution time distribution of the core modules in the proposed scheme. The sparse channel reconstruction module based on the SMO algorithm consumes the most time, accounting for 57.3% of the total overhead. If a further increase in positioning response speed is required, this module should be prioritized for optimization.

### 4.3. Computational Complexity and Real-Time Feasibility Analysis

Although Section 4.2 quantitatively evaluates the processing time of a single positioning task based on simulations, pure software-based simulations cannot fully demonstrate the system’s potential for real-time deployment due to the computational constraints of underlying hardware in practical applications. To improve interpretability and reproducibility for implementation, this section introduces an evaluation framework: theoretical complexity derivation—floating-point operations (FLOPs) calculation—hardware compute mapping. Specifically, we first derive the computational complexity of each module to identify the dominant factors of system latency and their dependence on key parameters. Next, we quantify this complexity into the floating-point operations (FLOPs) required for a single positioning task under default settings. Finally, we map the FLOPs to a representative microcontroller commonly used in VLP and estimate the processing latency on that platform. This provides quantitative evidence for real-time deployment feasibility.

As illustrated in the system architecture in Section 4.2, the online positioning process at the receiver mainly includes: signal preprocessing and observation vector generation, SMO-based sparse channel reconstruction and LOS power extraction, point classification and region-threshold matching, height estimation, and 3D coordinate solving based on LS. For complexity analysis, we define the following symbols (default values are given in Table 1): $N_{\mathrm{LED}}$ is the number of LEDs used for localization; $N_{p}$ is the length of one pilot symbol; $N_{1}$ is the number of pilot symbols sent by a single LED; $N_{g}$ is the cyclic prefix length; $L_{\max}$ is the number of samples corresponding to the maximum delay spread; *K* is the number of SMO iterations. According to the convergence process in Figure 7e, the proposed SMO algorithm typically converges within about 10 iterations.

For the pilot symbols of a single LED, FFT is performed on $N_{1}$ pilot symbols. Each FFT has a complexity of $O(N_{p}\log N_{p})$. Frequency-domain averaging is a pointwise accumulation and can be treated as a linear term $O(N_{1}N_{p})$. One IFFT is then performed. Therefore, the complexity of observation vector generation can be written as follows:

(16) $$C_{\mathrm{obs}}=O\;\left(N_{\mathrm{LED}}\cdot \left(N_{1}\cdot N_{p}\log N_{p}+N_{p}\log N_{p}+N_{1}\cdot N_{p}\right)\right)$$

This can be further simplified as

(17) $$C_{\mathrm{obs}}=O\;\left(N_{\mathrm{LED}}\cdot \left(N_{1}+1\right)\cdot N_{p}\log N_{p}\right)$$

During the SMO-based sparse channel reconstruction, the observation model is defined in Equation (10). The sensing matrix Φ is a Toeplitz matrix built from the pilot sequence, with dimensions M×N, where $M=N_{p}$ and $N=L_{\max}$. Although SMO introduces innovations within the OMP framework, the main computation comes from correlation search, support-set update and LS projection. At the *k*-th iteration, correlation search has a complexity of O(MN). After the support set is updated, a LS estimation is performed on the submatrix corresponding to the current support set, with a complexity of $O(Mk^{2})$. Therefore, for a single LED link, the total complexity of SMO over *K* iterations is

(18) $$C_{\mathrm{SMO}}=\sum_{k=1}^{K}\left(O\left(MN\right)+O\left(Mk^{2}\right)\right)=O\left(KMN\right)+O\;\left(M\sum_{k=1}^{K}k^{2}\right)$$

Since $\sum_{k=1}^{K}k^{2}=O\left(K^{3}\right)$, we obtain

(19) $$C_{\mathrm{SMO}}=O\;\left(KMN+MK^{3}\right)$$

Further considering independent reconstruction for $N_{\mathrm{LED}}$ LED links, the overall complexity becomes

(20) $$C_{\mathrm{SMO},\mathrm{total}}=O\;\left(N_{\mathrm{LED}}\cdot \left(KMN+MK^{3}\right)\right)=O\;\left(N_{\mathrm{LED}}\left(KN_{p}L_{\max}+N_{p}K^{3}\right)\right)$$

The MPEC computation and reverse verification introduced by SMO mainly operate on the current support set. Their additional overhead is a lower-order term compared to the O(MN) complexity of the correlation search. Thus, they do not change the dominant order of OMP-based algorithms. This is also consistent with Figure 16d, where SMO is the dominant runtime module in the simulation. From a computational complexity viewpoint, the online stages of the SVM(point-classification) and SVR(height-estimation) mainly involve kernel evaluations and weighted sums over a small number of support vectors. Their complexity is significantly lower than the O(KMN) overhead of the SMO algorithm. Based on the comprehensive analysis above, the total computational complexity of the online positioning system can be written as follows:

(21) $$C_{\mathrm{total}}=O\;\left(N_{\mathrm{LED}}\cdot \left(N_{1}+1\right)\cdot N_{p}\log N_{p}\right)+O\;\left(N_{\mathrm{LED}}\cdot \left(KN_{p}L_{\max}+N_{p}K^{3}\right)\right)$$

Equation (21) indicates that when $N_{\mathrm{LED}}$, $N_{1}$ and $L_{\max}$ are fixed, the overall complexity mainly increases with $N_{p}$ and *K*. In our setting and in typical indoor scenarios, *K* usually converges in about 10 iterations. Therefore, the complexity mainly shows an increasing trend with $N_{p}$. This conclusion is consistent with the simulation results in Figure 16a.

To assess the feasibility of physical implementation, we substitute the system parameters from Table 1 into the aforementioned model. After discrete conversion, the required floating-point operations (FLOPs) for a single 3D positioning task are estimated to be around $1.6\times 10^{6}$. In practical engineering, we take STM32F407 (STMicroelectronics, Geneva, Switzerland), a microcontroller commonly used in VLP [25]. This chip integrates a Cortex-M4 core and a hardware floating-point unit, offering a theoretical peak performance of approximately 168 MFLOPS. If the proposed algorithm is deployed on this chip, the execution latency is 9.52 ms. Even conservatively doubling this time to account for additional overheads in actual embedded systems, the single positioning latency remains well under 20 ms. This demonstrates strong potential for real-time deployment in engineering applications.

## 5. Conclusions

This paper proposes a novel SMO algorithm based on sparse channel reconstruction to suppress multipath effects. By integrating this algorithm with intelligent models, we develop a high-precision indoor VLP scheme. The scheme is designed to address the limitations of existing methods, including significant positioning errors due to multipath interference, the challenge of extending to 3D scenarios and poor adaptability to dynamic environments. Furthermore, it develops a full-process 3D positioning architecture featuring signal design, region adaptation, multipath suppression, height estimation and coordinate prediction. Simulation results demonstrate the comprehensive advantages of the proposed scheme. In 2D scenarios, the overall RMSE is 6.3cm, with the center area RMSE reaching only 2.7cm, representing an accuracy improvement of over 78% compared to the traditional LS method. In 3D scenarios, the average positioning error for 1.0m, 1.5m and zigzag planes is kept within 10cm. The average error for dynamic trajectories is less than 9cm, achieving centimeter-level precision. In terms of computational efficiency, the total latency for a single positioning task is controlled within 18.7ms with a pilot symbol length of 64. The system achieves a success rate of over 85% at a positioning update rate of 20Hz. By balancing centimeter-level accuracy with low-latency execution and low computational complexity, this scheme is well-suited for dynamic indoor positioning and shows significant potential for real-time deployment.

Based on the proposed VLP scheme, future research can be further conducted by exploring the synergistic transmission mechanism with visible light communication systems to achieve the integrated “communication–positioning” design.

## Figures and Tables

**Table 1:** Simulation parameters.

Parameter	Value
Room size (L×W×H)/m × m × m	5×5×3
Power of each LED bulb/W	4
FOV of PD $\psi_{c}$/(°)	70
Effective area of PD *A*/cm^2^	1
Half power angle of LED $\Phi_{1/2}$/(°)	70
Gain of optical filter $T_{s}\left(\varphi \right)$	1
Gain of concentrator g(φ)	1
Ceiling, wall, and floor reflectance	0.8, 0.8, 0.3
PD responsivity A/W	0.53
Minimum operating current $I_{L}$/A	0.05
Maximum operating current $I_{H}$/A	1
Background current $I_{bg}$/μA	5100
Scaling factor α	0.19
DC bias $B_{DC}$	0.365
Length of CP $N_{g}$	16
Number of pilot symbols	128
Length of pilot symbol $N_{p}$	64
Received sample interval/ns	2
Maximum delay spread/ns	128
System sampling rate $F_{s}$/MHz	500
Nyquist bandwidth $B_{\mathrm{Nyquist}}$/MHz	250
FFT size $N_{\mathrm{FFT}}$	64
Subcarrier spacing Δf/MHz	7.8125
Data symbol modulation scheme	16QAM
Number of data subcarriers $N_{\mathrm{data}}$	31
Actual occupied bandwidth $B_{\mathrm{occ}}$/MHz	242.1875
Data rate $R_{d}$/Mbps	775

**Table 2:** Comparison of RMSE for four different positioning methods.

Method	Entire Room RMSE/cm	Inner Area RMSE/cm	Edge Area RMSE/cm
Benchmark method 1	30.3	15.2	35.3
Benchmark method 2	22.6	13.9	30.1
Benchmark method 3	17.0	12.7	29.3
Proposed method	6.3	2.7	8.1

**Table 3:** Accuracy and precision metrics in different regions (2D).

Area	RMSE/cm	Average Error/cm	Standard Deviation/cm	Normalized Std Deviation
Inner Area	2.7	2.62	0.65	0.248
Entire Room	6.3	6.04	1.79	0.296
Edge Area	8.1	7.64	2.70	0.353

**Table 4:** Performance comparison of height estimation models based on intelligent algorithms.

Artificial Intelligence Algorithm	Average Height Estimation Error (cm)
Linear Regression	23.58
SVR	10.17
PSO-SVR	1.39
SSA-SVR	1.25
ELM	3.67

**Table 5:** Average point classification accuracy for 0.5 m, 1.0 m and 1.5 m planes.

Different Data Planes	Average Point Classification Accuracy
0.5 m	99.41%
1.0 m	100%
1.5 m	100%

**Table 6:** Accuracy and precision metrics in different regions (3D).

Area	RMSE/cm	Average Error/cm	Standard Deviation/cm	Normalized Std Deviation
Inner Area	6.17	5.94	1.67	0.281
Edge Area	9.69	9.10	3.33	0.366
Corner Area	11.81	10.86	4.64	0.427

## Data Availability

The data presented in this study are available on request from the corresponding author. The data are not publicly available due to secrecy restriction.

## Abbreviations

The following abbreviations are used in this manuscript:

ANN	Artificial Neural Network
AOA	Angle of Arrival
AWGN	Additive White Gaussian Noise
CDF	Cumulative Distribution Function
CIR	Channel Impulse Response
CNN	Convolutional Neural Network
CP	Cyclic Prefix
DCO-OFDM	Direct Current Biased Optical Orthogonal Frequency Division Multiplexing
FFT	Fast Fourier Transform
IFFT	Inverse Fast Fourier Transform
ISI	Intersymbol Interference
LOS	Line-of-Sight
LS	Least Squares
NLOS	Non-Line-of-Sight
OMP	Orthogonal Matching Pursuit
PD	Photoelectric Detector/Photodetector
RMSE	Root Mean Square Error
RSSI	Received Signal Strength Indicator
SMO	Spatial Adaptive–Main-Path Energy Constraint–Orthogonal Matching Pursuit (Shortened)
SSA	Sparrow Search Algorithm
SVM	Support Vector Machine
SVR	Support Vector Regression
TDM	Time Division Multiplexing
VLC	Visible Light Communication
VLP	Visible Light Positioning
